# JAK-STAT Pathway Inhibition Partially Restores Intestinal Homeostasis in *Hdac1*- and *Hdac2*-Intestinal Epithelial Cell-Deficient Mice

**DOI:** 10.3390/cells10020224

**Published:** 2021-01-23

**Authors:** Alexis Gonneaud, Naomie Turgeon, Francois-Michel Boisvert, Francois Boudreau, Claude Asselin

**Affiliations:** Département D’immunologie et Biologie Cellulaire, Pavillon de Recherche Appliquée Sur le Cancer, Faculté de Médecine et Des Sciences de la Santé, Université de Sherbrooke, Sherbrooke, QC J1E 4K8, Canada; Naomie.Turgeon@USherbrooke.ca (N.T.); Francois.Michel.Boisvert@USherbrooke.ca (F.-M.B.); Francois.Boudreau@USherbrooke.ca (F.B.); Claude.Asselin@USherbrooke.ca (C.A.)

**Keywords:** intestinal epithelial cell, HDAC, proteome, transcriptome, organoid, STAT3, tofacitinib

## Abstract

We have previously reported that histone deacetylase epigenetic regulator *Hdac1* and *Hdac2* deletion in intestinal epithelial cells (IEC) disrupts mucosal tissue architecture and barrier, causing chronic inflammation. In this study, proteome and transcriptome analysis revealed the importance of signaling pathways induced upon genetic IEC-*Hdac1* and *Hdac2* deletion. Indeed, Gene Ontology biological process analysis of enriched deficient IEC RNA and proteins identified common pathways, including lipid metabolic and oxidation–reduction process, cell adhesion, and antigen processing and presentation, related to immune responses, correlating with dysregulation of major histocompatibility complex (MHC) class II genes. Top upstream regulators included regulators associated with environmental sensing pathways to xenobiotics, microbial and diet-derived ligands, and endogenous metabolites. Proteome analysis revealed mTOR signaling IEC-specific defects. In addition to mTOR, the STAT and Notch pathways were dysregulated specifically in jejunal IEC. To determine the impact of pathway dysregulation on mutant jejunum alterations, we treated mutant mice with Tofacitinib, a JAK inhibitor. Treatment with the inhibitor partially corrected proliferation and tight junction defects, as well as niche stabilization by increasing Paneth cell numbers. Thus, IEC-specific histone deacetylases 1 (HDAC1) and 2 (HDAC2) support intestinal homeostasis by regulating survival and translation processes, as well as differentiation and metabolic pathways. HDAC1 and HDAC2 may play an important role in the regulation of IEC-specific inflammatory responses by controlling, directly or indirectly, the JAK/STAT pathway. IEC-specific JAK/STAT pathway deregulation may be, at least in part, responsible for intestinal homeostasis disruption in mutant mice.

## 1. Introduction

The intestinal epithelium interfaces the luminal microbiota and the mucosal immune system in a perpetual vigil, acting as a sensor of, and responder to, their respective signals [1,2]. The intestinal epithelium is unceasingly regenerated from crypt-residing intestinal stem cells (ISC) which produce transit-amplifying progenitor cells [3]. Upon their migration along the crypt-villus axis, these cells adopt differentiation programs to produce enterocyte and secretory cell lineages with complementary roles in establishing selective barrier function. Intestinal epithelial differentiated cells include enterocytes, with essential roles in nutrient absorption and digestion, in the establishment of the intestinal selective barrier through tight junctions and anti-bacterial gene expression, as well as immunoglobulin transport [4]. Goblet cells participate in barrier formation by secreting mucins as well as several anti-microbial proteins, and by acting as a conduit delivering luminal products to mucosal antigen-presenting cells [5]. Paneth cells, in addition to carving microbiota configuration and protecting the epithelium through secretion of anti-microbial proteins, regulate the stem cell niche environment [6]. Different types of hormone-secreting enteroendocrine cells display many roles, from systemic glucose homeostasis to mucosal immune responses [7,8]. Both ISC renewal and cell lineage specification depend on the coordinated action of various signaling pathways, including the Wnt, Notch and BMP pathways [3]. However, environmental as well as immune modifications along with genetic predispositions may overturn this delicate balance, leading to improper innate and adaptive intestinal inflammatory reactions, as observed in inflammatory bowel diseases (IBD) [9].

In recent years, a potential role of epigenetics in IBD, as receiver and transmitter of environmental changes, has emerged [10]. Indeed, some studies have uncovered IBD genetic susceptibility loci with epigenetic signatures associated with regulatory regions [11,12]. Lysine acetylation is one epigenetic mark controlling gene expression. While the acetyltransferase-dependent transfer of acetyl groups from acetyl-CoA on histones and other proteins is usually associated with gene expression, removal of the acetyl group by HDAC leads to gene repression. Acetylation is regulated in part by compartment-specific cellular acetyl-CoA levels responding to different environmentally induced metabolic changes [13]. In addition, both endogenous metabolites, such as β-hydroxybutyrate [14], as well as exogenous metabolites, such as the microbiota-produced butyrate [15], alter histone acetylation by inhibiting HDAC activity. Among the four HDAC classes, class I deacetylase HDAC1 and HDAC2, as part of multiprotein complexes, regulate gene expression as well as DNA replication and DNA repair [16,17]. While murine *Hdac1* deletion is developmentally lethal, *Hdac2* deletion results in heart defect-related death after birth. In contrast, most tissue-specific *Hdac1* and *Hdac2* individual or compound deletions have revealed both compensatory and distinct roles in tissue homeostasis and differentiation. Indeed, while tissue-specific *Hdac1* or *Hdac2* deficiencies lead to modest tissue alterations, deletion of both genes disrupts proliferation and differentiation in most tissues [16,17]. In the intestine, we have shown that villin-Cre-mediated *Hdac1* and *Hdac2* deletion disrupts IEC barrier function and differentiation, leading to chronic colonic inflammation [18]. Of note, intestinal tissue disruption in mutant mice is accompanied by simultaneous activation of the enterocyte-secretory cell lineage regulating Notch pathway, of the inflammation and mucosal healing supporting STAT3 pathway [19], and of the growth and metabolism-regulating mTOR pathway [20].

In this report, we used IEC-specific *Hdac1* and *Hdac2* deletion to determine the intrinsic jejunal IEC responses dependent upon HDAC1 and HDAC2, by transcriptome and proteome analysis. We first confirmed the importance of HDAC1 and HDAC2 for organoid growth, with both in vivo and inducible *Hdac1* and *Hdac2* deletion models. Omics data revealed that HDAC1 and HDAC2 control metabolic and environmental sensing pathways, and that IEC-specific deletion leads to improper STAT3, Notch and mTOR activation in IEC. We then assessed the importance of the STAT pathway in regulating both mucosal inflammation as well as disrupted Notch and mTOR pathways, by histological and immunostaining in mutant mice treated with a JAK/STAT pathway inhibitor, Tofacitinib. Our results show that the mutant phenotype is dependent upon STAT activation, as Tofacitinib treatment partially restores mucosal homeostatic responses, including proliferation and Paneth cell numbers. Thus, IEC-specific HDAC1 and HDAC2 support intestinal homeostasis by regulating survival and translation processes, as well as differentiation and metabolic pathways. They play an important role in the regulation of inflammatory responses by controlling, directly or indirectly, the JAK/STAT pathway. IEC-specific JAK/STAT pathway deregulation may be, at least in part, responsible for mucosal intestinal homeostatic disruption in mutant mice.

## 2. Material and Methods

### 2.1. Mice

*Hdac1* and *Hdac2* floxed mice [21] were crossed either with villin-Cre transgenic mice [22] to ensure IEC-specific *Hdac1* and *Hdac2* deletion, or with villin-Cre^ER^ transgenic mice [23], to obtain Tamoxifen-inducible IEC-specific *Hdac1* and *Hdac2* deletion, in a C57BL/6J X 129SV X CD1 background. Experiments were approved by the Institutional Animal Review Committee of the Université de Sherbrooke (protocol 360-14B), according to relevant guidelines and regulations. Genotypes were determined by using selected primers to amplify genomic DNA purified with the Spin Doctor genomic DNA kit (Gerard Biotech, Oxford, OH, USA), as before [18,24,25]. Three- to four-month-old wild-type and IEC-specific villin-Cre *Hdac1*^−/−^; *Hdac2*^−/−^ mice were injected intraperitoneally with 30 mg/kg of the pan-JAK inhibitor Tofacitinib (Cayman Chemical Company, Ann Arbor, MI, USA) [26], or with 10 mg/kg of the Notch inhibitor DBZ or Dimethyl Sulfoxide (DMSO), for five days (*n* = 3 for each group). To assess proliferation, mice were injected intraperitoneally with 10 mL/kg of bromodeoxyuridine (BrdU, Life Technologies, Grand Island, NY, USA) on day 6, for 2 h. Mice were killed on the sixth day. 

### 2.2. Enteroid Cultures

Jejunal crypts from three- to four-month-old wild-type and villin-Cre *Hdac1*^−/−^; *Hdac2*^−/−^ mice were fractionated by EDTA treatment, embedded in Matrigel and grown in ENR medium for five days, according to published methods [27,28], and as we have done before [29,30]. Jejunal crypts from control and *Hdac1*^−/−^; *Hdac2*^−/−^ villin-Cre^ER^ mice were isolated as above and treated with 0.5 µM of 4-hydroxy Tamoxifen (OHT)(Cayman Chemical Company) or DMSO for five days. Enteroids were passaged if needed. Enteroid phenotypes were assessed by microscopic image analysis with a Leica DC300 microscope (Leica Microsystems Inc., Richmond Hill, ON, Canada.) or a Cell Discoverer 7 microscope (magnification: 10×) (Zeiss, North York, ON, Canada). 

### 2.3. RNA Isolation and Quantitative Real-Time PCR (qPCR)

Jejunal IEC from three-month-old wild-type and villin-Cre *Hdac1*^−/−^; *Hdac2*^−/−^ mice were enriched by EDTA treatment. Jejunal IEC RNAs were isolated with the Rneasy kit (Qiagen, Montréal, QC, Canada). RNAs were quantified with a NanoDrop ND-1000 spectrophotometer (Thermo Fisher Scientific, Saint-Laurent, QC, Canada). cDNAs were synthesized from one µg RNA, with oligo dT15 primers and the Superscript II reverse transcriptase (Life Technologies Inc.). qPCR analysis was performed from 10 ng of cDNA, with the Brilliant III Ultra-fast SYBR Green qPCR Master Mix (Agilent Technologies, Saint-Laurent, QC, Canada) and gene-specific upstream and downstream oligonucleotides for stem cell marker *Lgr5*, secretory cell differentiation marker *Atoh1*, goblet cell marker *Muc2*, *Tff3* and *Zg16*, Paneth cell marker *Cryptdin*, *Lys2 and Defa24*, enterocyte marker *Sis*, *Slc15a1*, *Alpi* and *Fabp2*, enteroendocrine cell marker *ChgA*, proliferation marker *Ccnd1*, MHC II genes *Cd74*, *H2Aa*, *H2Ab1*, *H2Eb1*, *H2Dma*, *H2Dmb1*, *H2D* and *Ciita*, as well as *Reg3b*, *Reg3g* and *Jag2*, and the *Pbgd* loading control (Supplementary Table S1 for a list of primers). cDNA amplification was performed with a 10 min cycle at 95 °C, followed by 40 cycles of 10 s at 95 °C, 10 s at 60 °C and 20 s at 72 °C, on a Corbett RotorGene instrument (Qiagen/Corbett Research, Montréal, QC, Canada). Relative RNA amounts were determined by comparing to *Pbgd* amplification (*n* = 4–5).

### 2.4. RNA-Seq Analysis

Jejunal IEC from three-month-old wild-type and villin-Cre *Hdac1*^−/−^; *Hdac2*^−/−^ mice were enriched by EDTA treatment. Jejunal IEC RNAs were isolated with the ToTALLY RNA™ (Thermo Fisher Scientific) and Rneasy kits (Qiagen) (*n* = 3). Samples with RNA integrity number over 6.5 were selected with a 2100 Bioanalyser (Agilent Technologies). cDNA libraries were prepared and the transcriptome was determined with the Illumina HiSeq4000 PE100 sequencing system (Illumina, San Diego, CA, USA) at the McGill University and Génome Québec Innovation Center. Sequences were aligned with the Star 2.4.0.1 software package based on the genome reference Mus_musculus:GRCm38. Differentially expressed genes were identified with DESeq adjusted *p*-value ≤ 0.05 [31]. Two-fold increased or decreased transcripts between control and *Hdac1*^−/−^; *Hdac2*^−/−^ IEC were selected for additional bioinformatics analysis. RNA-Seq data have been deposited in the Gene Expression Omnibus database (GSE158522).

### 2.5. Proteome Analysis

Jejunal IEC from three-month-old wild-type and villin-Cre *Hdac1*^−/−^; *Hdac2*^−/−^ mice were enriched by EDTA treatment. IEC were lysed in TEAB buffer (100 mM triethylammonium bicarbonate, 10% SDS) (*n* = 3). Amounts of 50 µg of proteins were reduced with tris(2-carboxyethyl)phosphine for one hour at 55 °C, and alkylated with 375 mM iodoacetamide for 30 min in the dark. Proteins were precipitated with acetone for 4 h at −20 °C, and suspended in TEAB buffer and trypsin for overnight digestion at 37 °C. After digestion, peptides were labeled with isobaric chemical Tags of 126 to 128 Da (controls) and 129 to 131 Da (mutants), for one h at room temperature (Tandem Mass Tag Reagents (TMT), Thermo Fisher Scientific). The reaction was stopped by adding 5% hydroxylamine for 15 min. Labeled peptides were mixed and washed on C18 columns, according to the manufacturer’s instructions. Samples were then separated by liquid phase chromatography and MS/MS mass spectrometry. Peptides were separated with an ultimate U3000 nanoflow LC-system (Dionex Corporation, Sunnyvale, CA, USA). The HPLC system was coupled to an OrbiTrap Q Exactive, via an EasySpray source. The following parameters were used, as suggested by the manufacturer. For MS: Resolving power (FWHM at m/z 200), 70,000; AGC target, 3 × 10^6^; Maximum injection time (ms), 50; Scan range (m/z), 375–1400. For MS/MS: Resolving power (FWHM at m/z 200), 35,000; AGC target, 1 × 10^5^; Maximum injection time (ms), 100; Isolation width (Th), 0.7 or 1.2; Normalized collision energy (NCE), 32 (Thermo Fisher Scientific). Data were acquired with the Excalibur software. Protein identification, as well as TMT-labeled peptide quantitation, were performed with the MaxQuant version 1.5.2.8 software [32] and the Uniprot mouse protein database, as we have done before [29]. The false discovery rate (FDR) was adjusted to 5%. Then, 50% increased or decreased proteins between control and *Hdac1*^−/−^; *Hdac2*^−/−^ IEC were selected for additional bioinformatics analysis. Mass spectrometry proteomic data were deposited to the ProteomeXchange Consortium through the PRIDE partner repository with the dataset identifier PXD (PXD022558) (http://proteomecentral.proteomexchange.org - PRIDE (EMBL-EBI, Cambridge, UK).

### 2.6. Bioinformatics Analysis

Transcriptome and proteome data were used to classify genes according to Gene Ontology with the Database for Annotation, Visualization and Integrated Discovery software (DAVID 2.0) [33] and the Ingenuity Pathway Analysis software (IPA, Qiagen). Pathway activation (Upstream Regulator Analysis (URA), z-score) was predicted from the variations between samples and the altered biological processes [34]. 

### 2.7. Histological Analysis, Immunofluorescence and Immunocytochemistry

Wild-type and *Hdac1*^−/−^; *Hdac2*^−/−^ villin-Cre^ER^ enteroids treated with or without 0.5 µM of 4-hydroxy Tamoxifen for five days were used (*n* = 3). Paraformaldehyde-fixed enteroids were mixed with Histogel (Thermo Fisher Scientific) before paraffin inclusion. Nuclei were stained with DAPI when needed. To assess DNA damage by immunofluorescence, paraffin-embedded enteroid sections were labeled with a primary rabbit antibody against phospho-histone γH2A.X (sc-101696) (1:500, Santa Cruz Biotechnology, Santa Cruz, CA, USA) and F(ab’) rabbit IgG (H + L) (Alexa Fluor 568) preadsorbed secondary antibodies (Abcam, Cambridge, MA, USA), as previously done [30].

Wild-type and villin-Cre *Hdac1*^−/−^; *Hdac2*^−/−^ jejuna, from mice treated with or without Tofacitinib, were fixed in 4% paraformaldehyde and enclosed in paraffin. Four µm-sections were stained with hematoxylin and eosin for histological analysis, and with Best’s Carmine for Paneth cell staining. Proliferation was determined by staining with fluorescein-conjugated mouse antibody against bromodeoxyuridine (BrdU) (1:50, BMC 9318, Roche Diagnostics, Laval, QC, Canada). BrdU positive nuclei and Paneth cells were counted with Cell Profiler 3.15 [35]. Sections were rehydrated with graded ethanol series containing 100, 95, 80 and 70% xylene, and then boiled for 12 min in 10 mM citric acid for immunofluorescence, or boiled for 20 min in a 10 mM Tris pH 9.0, EDTA 1 mM, 0.05% Tween-20 solution for immunocytochemistry. Sections were blocked in a PBS solution containing 0.1% BSA and 0.2% Triton X-100 for 45 min. For immunofluorescence experiments, sections were incubated with a rabbit antibody against Claudin-3 (341700) (1:200, Invitrogen) or a goat antibody against lysozyme (sc-27958) (1:500, Santa Cruz Biotechnology). Primary antibodies were recognized with secondary Alexa Fluor 568 goat anti-rabbit IgG (H + L) or Alexa Fluor 555 donkey anti-goat IgG (H + L) (Life Technologies). For immunocytochemistry experiments, sections were incubated with rabbit antibodies against phospho-S6 ribosomal protein (#2211) (1:500, Cell Signaling, Whitby, ON, Canada), against Stat3 (#12640) (1:200, Cell Signaling) and against the T-cell marker CD3 (A0452) (1:500, Dako Canada, Burlington, ON, Canada), followed by labeling with EnVision + System-HRP (Dako Canada). Information about antibodies is included in Supplementary Table S2.

### 2.8. Western Blot Analysis

Jejunal IEC from three-month-old wild-type and villin-Cre *Hdac1*^−/−^; *Hdac2*^−/−^ mice were enriched by EDTA treatment. IEC were lysed in 1 X Laemmli buffer (62.5 mM Tris-HCl pH 6.8, 2% SDS, 10% glycerol) supplemented with protease and phosphatase inhibitors. Protein concentrations were determined with the Pierce BCA Protein Assay kit (Therrmo Fisher Scientific). Amounts of 15 µg of total proteins were separated on 4–12% SDS-polyacrylamide gels, and transferred on PVDF membranes (Roche Molecular Biochemicals). Membranes were incubated 1 h at room temperature or overnight at 4 °C with primary antibodies including rabbit anti-phospho-Stat3 (#9145, Cell Signaling), rabbit anti-Stat3 (#12640, Cell Signaling), rabbit anti-phospho-p38 (#4511, Cell Signaling), rabbit anti-claudin 3 (341700, Invitrogen), rabbit anti-cleaved Notch (#4147, Cell Signaling), rabbit anti-phospho-S6 (#4858, Cell Signaling) and GAPDH (#2118, Cell Signaling). Primary antibodies were recognized with secondary goat anti-rabbit antibodies (Life Technologies) before immune complex detection with Amersham ECL^TM^ Western blotting detection reagents (GE Healthcare, Mississauga, ON, Canada).

### 2.9. Statistical Analysis

Data are expressed as the mean ± SEM. Groups were compared by Student’s *t*-test (unpaired), or one-way ANOVA with Tukey multiple comparison test.

## 3. Results and Discussion

**Figure 1:**
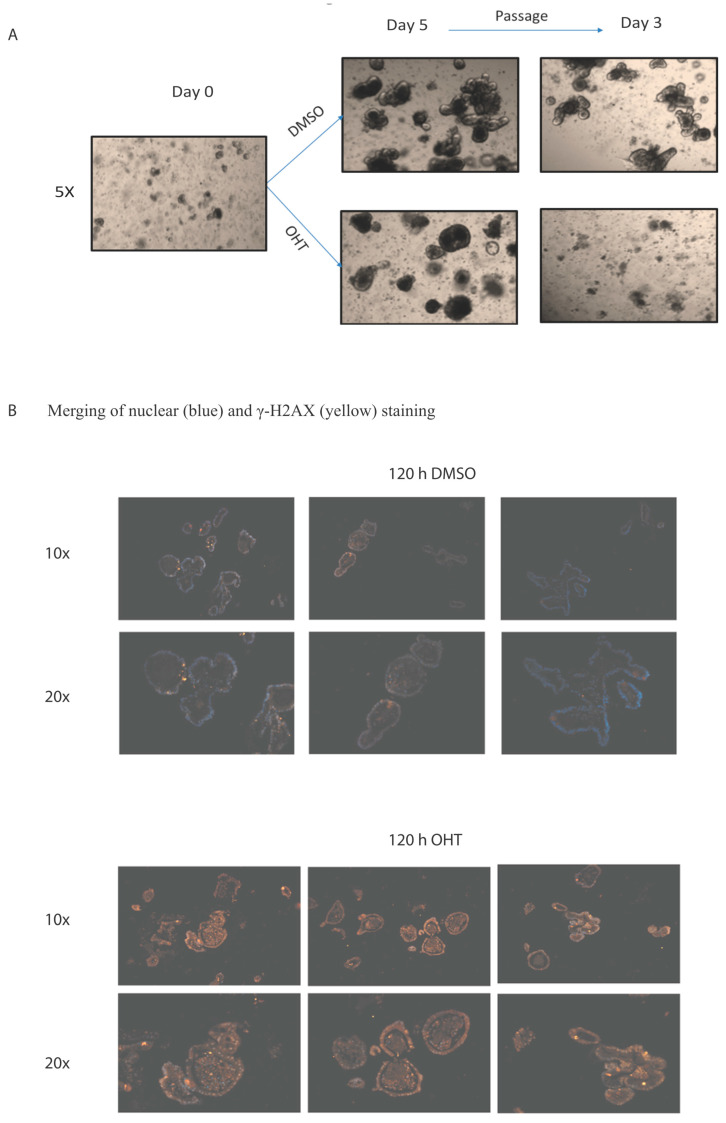
Enteroid growth is disrupted after inducible *Hdac1* and *Hdac2* deletion. (**A**) Representative micrographs of 5-day DMSO or tamoxifen (OHT) treated control or *Hdac1*^−/−^; *Hdac2*^−/−^ villin-Cre^ER^ enteroids (*n* = 3) and three days after passage. Magnification: 5×. (**B**) Representative images of merged 5-day DMSO or tamoxifen (OHT) treated control or *Hdac1*^−/−^; *Hdac2*^−/−^ villin-Cre^ER^ enteroids stained with an antibody against phospho-γ-H2AX and with DAPI for nuclear staining (*n* = 3). Magnification: 10×, 20×.

We have previously observed that IEC-specific dual deletion of *Hdac1* and *Hdac2* led to IEC differentiation and proliferation alterations, as well as chronic inflammation in the colon [18]. To determine whether HDAC1 and HDAC2 are both needed for IEC growth and survival, we generated enteroid cultures from villin-Cre dual *Hdac1*^−/−^; *Hdac2*^−/−^ murine crypts. After five days of culture, growth was stopped in all enteroids, with the absence of budding (*n* = 3, Supplementary Figure S1). Attempts to passage the enteroids were unsuccessful. This suggests that during enteroid growth, deletion of the remaining *Hdac1* or *Hdac2* allele occurs, leading to cell growth inhibition and death. We then produced enteroids from crypts isolated from villin-Cre^ER^ dual *Hdac1*^−/−^; *Hdac2*^−/−^ mice. As opposed to control enteroids, villin-Cre^ER^ enteroids treated for 5 days with Tamoxifen displayed enteroid structure alterations, reduction in budding and growth arrest (Figure 1A), as well as increased phospho-γ-H2AX staining, a marker of DNA damage (Figure 1B). Thus, growth arrest of *Hdac1*^−/−^; *Hdac2*^−/−^ KO enteroids may be due to a combination of chromatin destabilization and accumulation of DNA damage, which may lead to organoid death. Indeed, HDAC1 and HDAC2 are both needed to regulate DNA repair and the double-strand break-dependent DNA damage response [36]. In agreement with this, we have previously observed increased phospho-γ-H2AX staining in *Hdac1*- or *Hdac2*-depleted enteroids, suggesting again augmented sensitivity to DNA damage upon DNA replication [30]. Our results, with in vivo and inducible deletion organoid models, confirm those of Zimberlin et al. [37], that both *Hdac1* and *Hdac2* are necessary for organoid growth.

The question remains about the difference between the death of organoids deleted for HDAC1 and HDAC2 and living mutant mice; which pathways are implicated in maintaining mutant mice alive? We have previously shown by microarray analysis of total IEC-specific *Hdac1* and *Hdac2* deleted colonic RNAs the presence of chronic intestinal inflammation [18]. To determine the IEC-intrinsic effect of *Hdac* deletion in the jejunum of mutant mice, we verified the global transcriptome and proteome of EDTA-enriched jejunal cells from wild-type and villin-Cre *Hdac1*^−/−^; *Hdac2*^−/−^ mice. RNA-Seq analysis identified 1904 genes with increased expression (more than 2-fold) and 1682 genes with decreased expression (less than 0.5-fold) in dual *Hdac*-deficient jejunal cells (DESeq adjusted *p*-value ≤ 0.05), representing respectively 9.7% and 8.6% of total identified genes (Supplementary Table S3). Mass spectrometry proteomic analysis of differentially labeled TMT proteins revealed 332 proteins increased (more than 1.5-fold) and 423 proteins decreased (less than 0.75-fold) in dual *Hdac*-deficient jejunal cells (FDR 5%), representing respectively 10.3% and 13% of the total detected proteins (Supplementary Table S4). 

**Figure 2:**
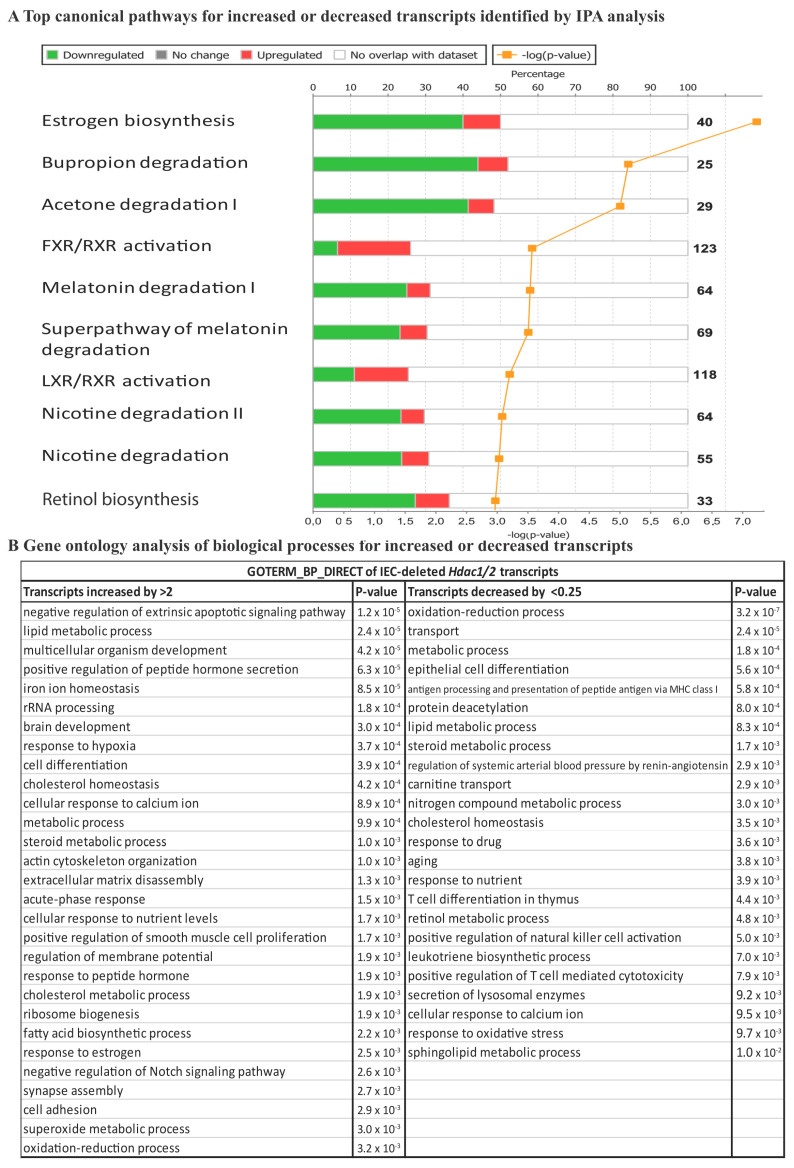
RNA expression modifications in enriched *Hdac1* and *Hdac2* deleted jejunal intestinal epithelial cells. (**A**) Top canonical pathways for increased or decreased transcripts were identified by IPA analysis. (**B**) Gene ontology analysis of biological processes for increased or decreased transcripts was performed with the DAVID 2.0 EASE Score. *p*-value < 0.05; fold-change: more than 2-fold or less than 0.5-fold.

**Figure 3:**
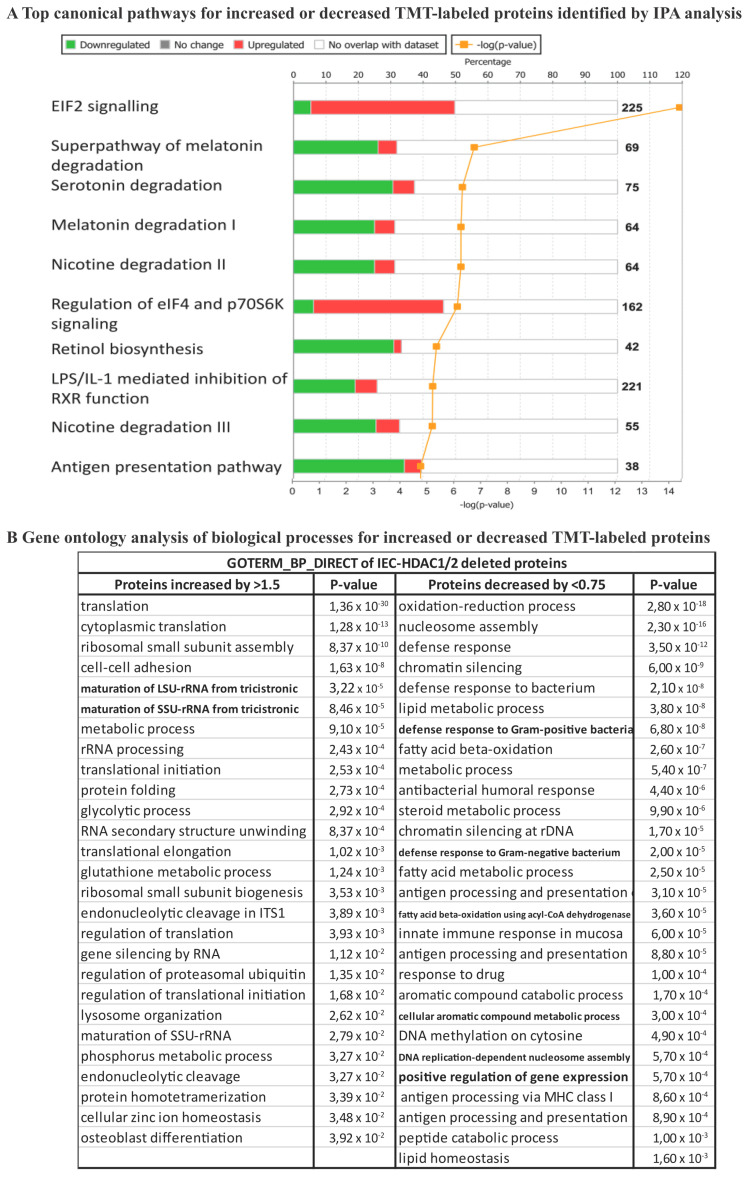
Protein expression modifications in enriched *Hdac1* and *Hdac2* deleted jejunal intestinal epithelial cells. (**A**) Top canonical pathways for increased or decreased TMT-labeled proteins were identified by IPA analysis. (**B**) Gene ontology analysis of biological processes for increased or decreased TMT-labeled proteins was performed with the DAVID 2.0 EASE Score. *p*-value < 0.05; fold-change: more than 1.5-fold and less than 0.75-fold.

Among the top canonical pathways, retinol biosynthesis, melatonin and nicotine degradation pathways were shared between the transcriptome and the proteome (Figure 2A and Figure 3A). Data analysis revealed estrogen biosynthesis, FXR/RXR and LXR/RXR activation as top canonical pathways for the transcriptome (Figure 2A), and EIF2 signaling, LPS/IL-1 mediated inhibition of RXR function and antigen presentation pathway for the proteome (Figure 3A). Activation of nuclear receptors such as FXR, is related to deregulation of lipid metabolism pathways, including oxidation of fatty acids [38], that may provide energy for IEC as well increased production of the acetyl donor, acetyl-CoA, the major regulator of histone acetylation. EIF2 signaling activation observed in the proteome analysis, but not the transcriptome analysis, confirms mTOR pathway activation associated with increased small intestine lengthening, observed in villin-Cre *Hdac1*^−/−^; *Hdac2*^−/−^ mice [18]. Gene Ontology analysis with DAVID identified common proteome and transcriptome biological processes, such as lipid metabolic process, metabolic process, cell adhesion, oxidation-reduction process and antigen processing and presentation, related to immune responses. It was observed that after HDAC inhibition in dendritic cells results in STAT3-regulated gene expression modifications. Downregulated STAT3-dependent targets are involved in immune effector processes and antigen processing/presentation, as we observed here. This suggests a direct interaction between HDAC activity and STAT3-targeted gene regulation [39]. Again, most deregulated pathways following *Hdac1* and *Hdac2* loss in IEC involve metabolic processes associated with acetyl-CoA production pathways. GO analysis identified specific transcriptome-associated biological processes, such as negative regulation of extrinsic apoptosis, multicellular organism development, epithelial cell differentiation and transport, and specific proteome-associated biological processes related to translation, defense response to bacteria and chromatin regulation (Figure 2B and Figure 3B). Disease and biological functions sorted by z-score highlighted downregulation of lipid-related functions at both RNA and protein levels (Supplementary Table S5). Top upstream regulators common to mutant transcriptome and proteome included CFTR, PPARA with associated regulators (ciprofibrate and ACOX), PPARG, HNF4A and IL10RA, as determined by IPA analysis (Supplementary Table S6). Thus, HDAC-dependent epigenetic modifications play an important role in controlling intrinsic and extrinsic pathways of intestinal homeostasis. Indeed, *Hdac* deletion results in consistent alterations in environmental sensing pathways to xenobiotics, microbial and diet-derived ligands, as well as endogenous cell metabolites.

**Figure 4:**
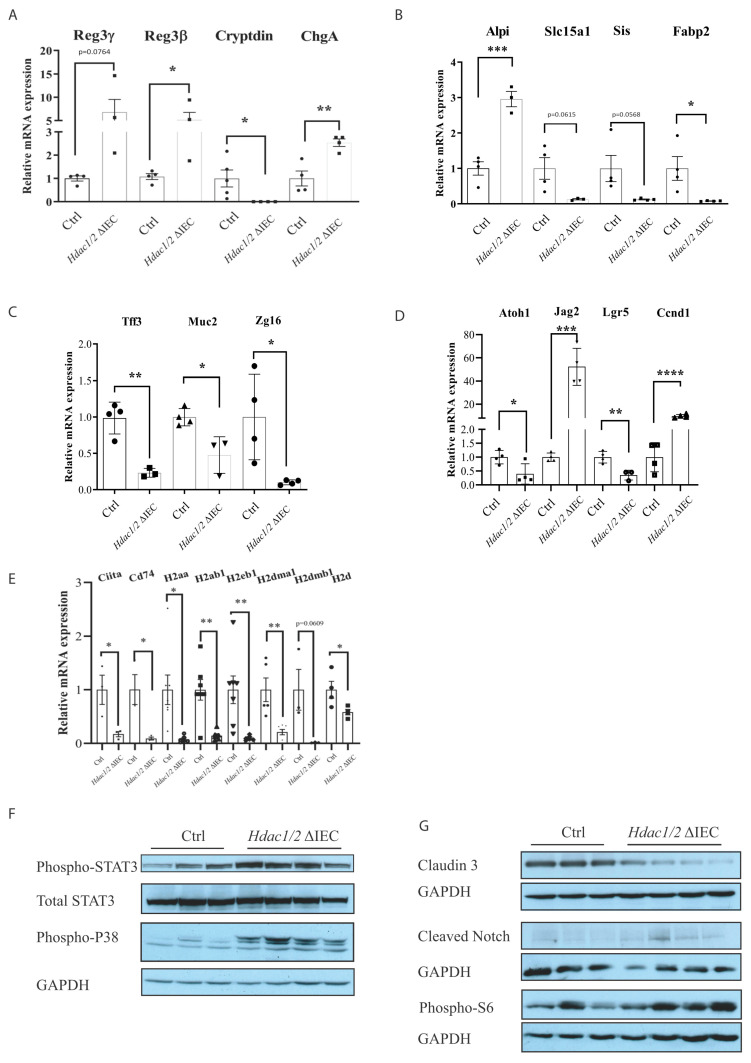
*Hdac1* and *Hdac2* deletion disrupts jejunal epithelial differentiation and MHC class II gene expression. Total RNA was purified from enriched control and *Hdac1*^−/−^; *Hdac2*^−/−^ jejunal IEC. Expression levels of secretory cell differentiation marker *Reg3g*, *Reg3b*, *Cryptdin* and *ChgA* (**A**), enterocyte cell marker *Alpi*, *Slc15a1*, *Sis* and *Fabp2* (**B**), goblet cell marker *Tff3*, *Muc2* and *Zg16* (**C**), differentiation, proliferation and stem cell marker *Atoh1*, *Jag2*, *Lgr5*, *Ccnd1* (**D**), as well as MHC II genes *Ciita*, *Cd74*, *H2aa*, *H2ab1*, *H2eb1*, *H2dma1*, *H2dmb1* and *H2d* (**E**) were measured by qPCR. Relative RNA amounts were determined by comparing to *Pbgd* amplification (*n* = 4–5). Results represent the mean ± SEM (* *p* ≤ 0.05; ** *p* ≤ 0.01; *** *p* ≤ 0.005; **** *p* ≤ 0.001). Protein extracts enriched from control and *Hdac1*^−/−^; *Hdac2*^−/−^ jejunal IEC were separated on 8% or 10% SDS-PAGE gels and transferred to PVDF membranes for Western blot analysis of phospho-STAT3, total STAT3 and phospho-p38 (**F**), or claudin 3, cleaved Notch and phospho-S6 (**G**). GAPDH was used as loading control for each membrane.

Previous microarray and QPCR analysis with total mucosal RNA preparations from mutant colons indicated an inflammatory state in mutant mice, associated with differentiation defects. Here, we measured by qPCR intrinsic gene expression changes in enriched *Hdac*-deleted IEC, without the mucosal portions. C-type lectin *Reg3β* and *Reg3γ* gene expression was increased (Figure 4A). This antimicrobial peptide family controls intestinal barrier processes, in part by controlling luminal bacterial content and by regulating innate as well as adaptive mucosal immune responses [40]. Paneth cell differentiation was altered, as exemplified by decreased expression of antimicrobial *Cryptdin* (Figure 4A), and of novel markers including the Krüppel-like transcription factor *Klf15* and the mucosal pentraxin *Mptx2* (respectively −2.781 and −2.26 (log_2_)) ([41]; Supplementary Table S3). Some enteroendocrine cell (EEC) hormone genes expressed in different EEC subsets were increased, such as *ChgA* encoding a neuroendocrine secretory protein (Figure 4A), and secretin (*Sct*), a pan-EEC marker (4.094 (log_2_)), or decreased (Gastric inhibitory peptide (*Gip*), −1.255 (log_2_); Glucagon (*Gcg*) −1.266 (log_2_)). Other subset-specific EEC genes, such as Neurotensin (*Nts*) and Somatostatin (*Sst*), were decreased (−1.31, −3.966 (log_2_), Supplementary Table S3) [7,41]. Enterocyte and goblet cell differentiation was altered (Figure 4B, 4C). Indeed, enterocyte-expressed *Slc15a1*, encoding the brush border intestinal hydrogen peptide cotransporter PepT1 [42], *Sis*, encoding the brush border sucrase–isomaltase and *Fabp2*, encoding a fatty acid protein involved in long-chain fatty acid uptake and transport, were decreased, while *Alpi*, an alkaline phosphatase gene with anti-inflammatory properties, namely through bacterial lipopolysaccharide detoxification, was increased [43,44] (Figure 4B). Goblet-expressed *Tff3*, encoding a repair-inducing protective Trefoil family secretory protein [45], *Muc2*, encoding a secreted mucin involved in mucous barrier formation, and *Zg16,* encoding a lectin-like protein acting as a bacterial segregator [46], were decreased (Figure 4C). Thus, while differentiation and function of the three main IEC types, namely enterocytes, secretory Paneth and goblet cells, are altered in knockout cells, a number of protective genes, including *Reg* and *Alpi* genes, are increased, and may play important roles to ensure tissue homeostasis.

We also observed decreased expression of *Atoh1*, encoding an IEC secretory lineage differentiation regulator, and *Lgr5*, encoding an R-spondin receptor involved in stem IEC maintenance, as well as increased expression of *Jag2*, encoding a Notch receptor activating ligand, and *Ccnd1*, encoding the cell cycle regulator Cyclin D1 (Figure 4D). Thus, gene expression results on isolated IEC confirm that IEC-specific *Hdac1* and *Hdac2* deletion alters Notch signaling and stem cell marker expression, as well as IEC proliferation [18]. 

As Omics analysis suggested defects in pathways related to antigen processing and presentation, we verified MHC class II gene expression (Figure 4E). Indeed, IEC act as antigen-presenting cells, in part by expressing MHC class II genes and regulators both in differentiated cells [47] and in stem cell subtypes [48]. Recent data suggest that IEC-specific MHC class II gene deletion leads to increased intestinal stem cell numbers, as well as IEC differentiation and T cell activation defects [48]. qPCR analysis showed reduced expression of a number of MHC Class II genes, as well as the MHC Class II *Ciita* transcriptional regulator and *Cd74*, a chaperone associated with MHC Class II [47]. Thus, HDAC may be important regulators of MHC class II IEC expression patterns which could affect both the mucosal immune responses and IEC barrier homeostasis.

We then assessed the intrinsic IEC effect of *Hdac* and *Hdac2* deletion on signal transduction pathways, by Western blot analysis of IEC-enriched protein extracts. Both STAT3 and p38 inflammatory signaling was induced in *Hdac*-deleted IEC, as shown by increasing STAT3 and p38 phosphorylated forms as opposed to control IEC (Figure 4F). Notch signaling was increased, as assessed by augmented levels of cleaved Notch (Figure 4G). Notch signalling activation not only maintains intestinal epithelial stem cells, but also regulates IEC progenitor differentiation into enterocytes while repressing the secretory differentiation pathway [49]. In addition, the mTOR kinase pathway was activated, as shown by increased levels of phospho-ribosomal S6 protein, a downstream target of S6 kinases (Figure 4G). mTOR may play a role in intestinal regeneration upon injury [50]. Intrinsic IEC mTOR pathway activation is thus associated with increased small intestinal growth in IEC-specific *Hdac1* and *Hdac2* deleted mice [18]. Finally, protein levels of CLDN3, a component of tight junctions regulating IEC polarity and barrier functions, and a marker of intestinal barrier integrity [51], were reduced (Figure 4G).

Thus, IEC-specific *Hdac1* and *Hdac2* deletion leads to activation of Notch, Stat3 and mTOR pathways involved in stemness, differentiation and inflammatory responses. Results obtained in different cell lines have uncovered crosstalk interactions between these pathways. For example, mTOR may phosphorylate and activate Stat3 [52]. A mTOR-Stat3-Notch pathway is involved in the control of murine embryonic fibroblast and human breast cancer cell line differentiation [53]. Notch receptor ligands may lead to Stat3 activation of Hes family members in neural stem cells [54]. Finally, Notch activation may induce mTOR activation during liver lipogenesis [55,56]. To determine the impact of these pathways on the mutant mouse phenotype, we focused on the Notch and Stat3 pathways. We treated mice respectively with Dibenzazepine (DBZ), a Notch/γ-secretase inhibitor, to block the Notch pathway [57], and Tofacitinib, a JAK kinase inhibitor, to block the JAK-STAT pathway [58].

As observed before, IEC-specific *Hdac1* and *Hdac2* deletion resulted in decreased secretory Paneth and goblet cell numbers, crypt elongation and impaired epithelial polarity, as evidenced by disrupted nuclear staining and localization ([18]; Supplementary Figure S2A). In contrast to control mice, DBZ treatment was lethal to some mutant mice. DBZ treatment increased the number of goblet cells in control mice. H and E staining of jejunal sections from surviving mutant mice revealed an increase in the number of goblet cells and eosinophilic granule-stained Paneth cells in the crypt. Thus, by blocking the Notch pathway, IEC secretory lineage differentiation was increased in both control and mutant mice However, it was quite evident that DBZ treatment intensified epithelial disruption in mutant mice. As Notch signaling is involved in maintaining stem cell pools, we have injected mice with BrdU and evaluated proliferation in intestinal crypts (Supplementary Figure S2B). As observed before, IEC-specific *Hdac1* and *Hdac2* deletion led to enhanced crypt proliferation and expansion. In contrast to control mice, DBZ treatment decreased proliferative cell numbers at the bottom of the crypts, with some proliferative cells maintained in the upper crypt region. Thus, IEC-specific *Hdac1* and *Hdac2* deletion renders mice more sensitive to Notch signaling alterations. Diversion of stem cells towards secretory cell differentiation may deplete the stem cell pool, thereby affecting epithelial maintenance in an injury-like setting, such as found in the mutant *Hdac1* and *Hdac2* deletion mouse mucosa.

**Figure 5:**
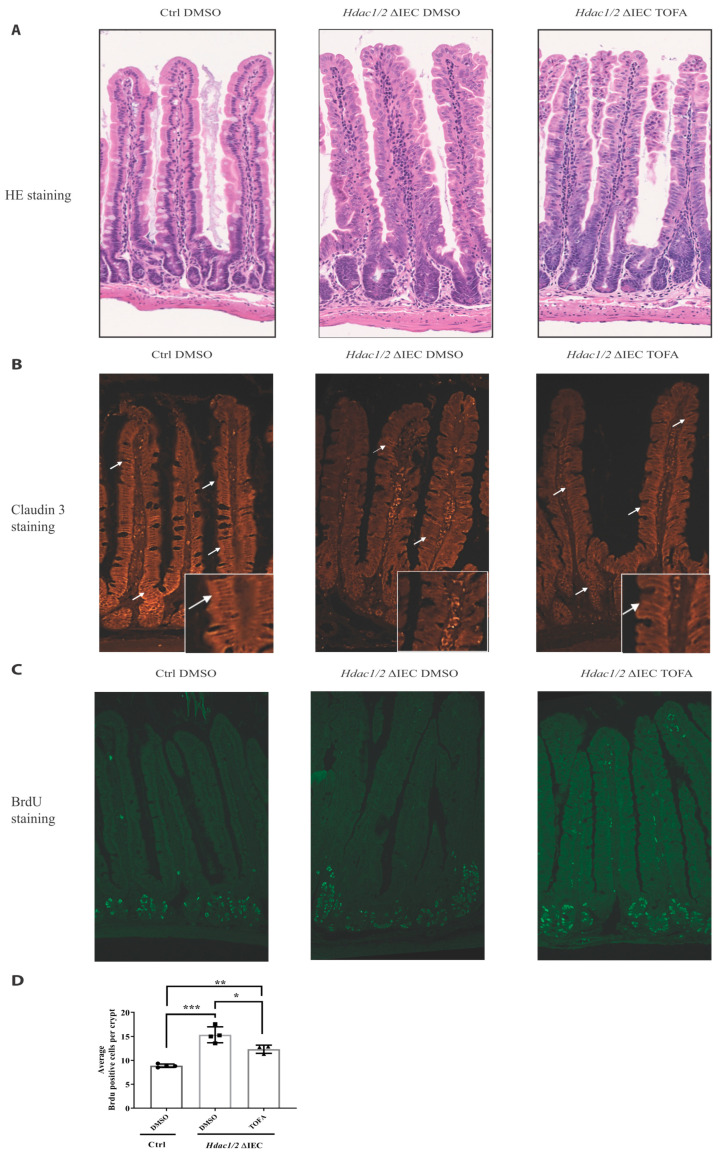
Tofacitinib treatment ameliorates intestinal architecture and decreases intestinal epithelial cell proliferation in villin-Cre *Hdac1* and *Hdac2* deficient mice. Jejunal tissue sections, from wild-type (Ctrl) and villin-Cre *Hdac1*^−/−^; *Hdac2*^−/−^ mice (*Hdac1/2* ΔIEC) treated without or with Tofacitinib (DMSO or TOFA) for five days, were stained with hematoxylin and eosin (HE) (**A**) (Magnification: 10×) or were stained with an antibody against claudin 3 (*n* = 3) (white arrows) (**B**) (Magnification: 20×). (**C**) Wild-type (Ctrl) and villin-Cre *Hdac1*^−/−^; *Hdac2*^−/−^ mice, treated without or with Tofacitinib (DMSO or TOFA) for five days, were injected with BrdU for 2 h. Jejunal tissue sections were stained with an antibody against incorporated BrdU. Magnification: 10×. (**D**) The average number of BrdU-labeled proliferative cells per jejunal crypts was measured (*n* = 3, 20–30 crypts each). Results represent the mean ± SEM (* *p* ≤ 0.05; ** *p* ≤ 0.01; *** *p* ≤ 0.005).

**Figure 6:**
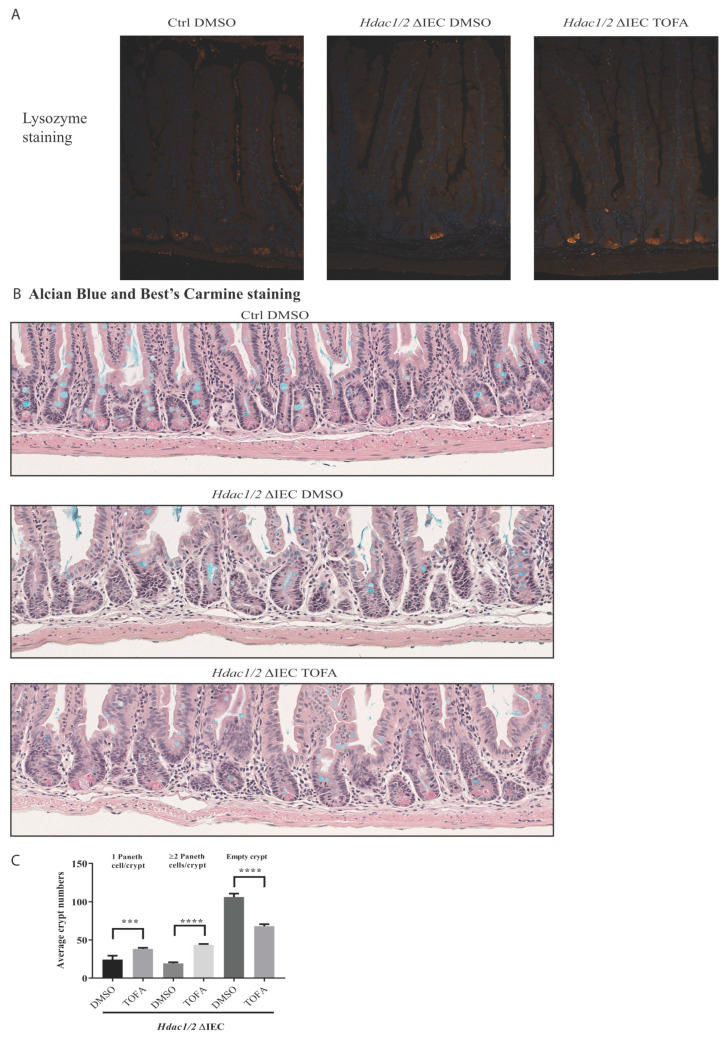
Tofacitinib treatment increases the number of Paneth cells in villin-Cre *Hdac1* and *Hdac2* deficient mice. Jejunal tissue sections, obtained from wild-type (Ctrl) as well as villin-Cre *Hdac1*^−/−^; *Hdac2*^−/−^ mice (*Hdac1/2* ΔIEC) treated without or with Tofacitinib (DMSO or TOFA) for five days, were stained with an antibody against lysozyme for Paneth cell staining (white arrows), and merged with DAPI nuclear staining (**A**), or with Best’s Carmine for Paneth cell staining (black arrows) and Alcian Blue for goblet cell staining (**B**). Magnification 10×. (**C**) The average number of crypts with none, or with one, two or more Paneth cells was compared between jejunal tissue sections from villin-Cre *Hdac1*^−/−^; *Hdac2*^−/−^ mice treated without or with Tofacitinib (DMSO or TOFA) (*n* = 3, 150 crypts each). Results represent the mean ± SEM (*** *p* ≤ 0.005; **** *p* ≤ 0.001).

To determine the importance of Stat3, a major pathway activated in dual IEC-specific *Hdac1* and *Hdac2*-deficient mice, we inhibited Stat signaling with Tofacitinib, a JAK inhibitor. As opposed to control mutant mice, Tofacitinib treatment led to partial restoration of nuclear basal epithelial localization, as assessed by H and E staining (Figure 5A). In control mice, immunofluorescence staining showed that CLDN3 was restricted to basolateral membranes in both jejunal upper crypt and villus compartments (Figure 5B), as observed in rat duodenal stained sections [59]. In mutant mucosa, in addition to decreased CLDN3 expression, CLDN3 was delocalized (Figure 5B). In contrast, Tofacitinib treatment restored both CLDN3 levels and basolateral localization, suggesting amelioration of barrier function. Finally, we assessed crypt cell proliferation after BrdU staining. While crypt cell proliferative numbers were enhanced in mutant mice, Tofacitinib treatment reduced the number of BrdU positive cells to intermediary levels, in comparison to control mice. (Figure 5C,D). This correlated with the appearance of Paneth cells that help to stabilize the crypt niche in mutant mice. While Tofacitinib treatment did not increase goblet cell numbers in mutant mice, as assessed by Alcian blue staining, Paneth cell numbers were increased, as shown by increased Lysozyme- and Best’s Carmine-stained cells in the crypt, as well as by increased numbers of crypts containing more than one Paneth cell, as opposed to non-treated mutants (Figure 6A–C).

**Figure 7:**
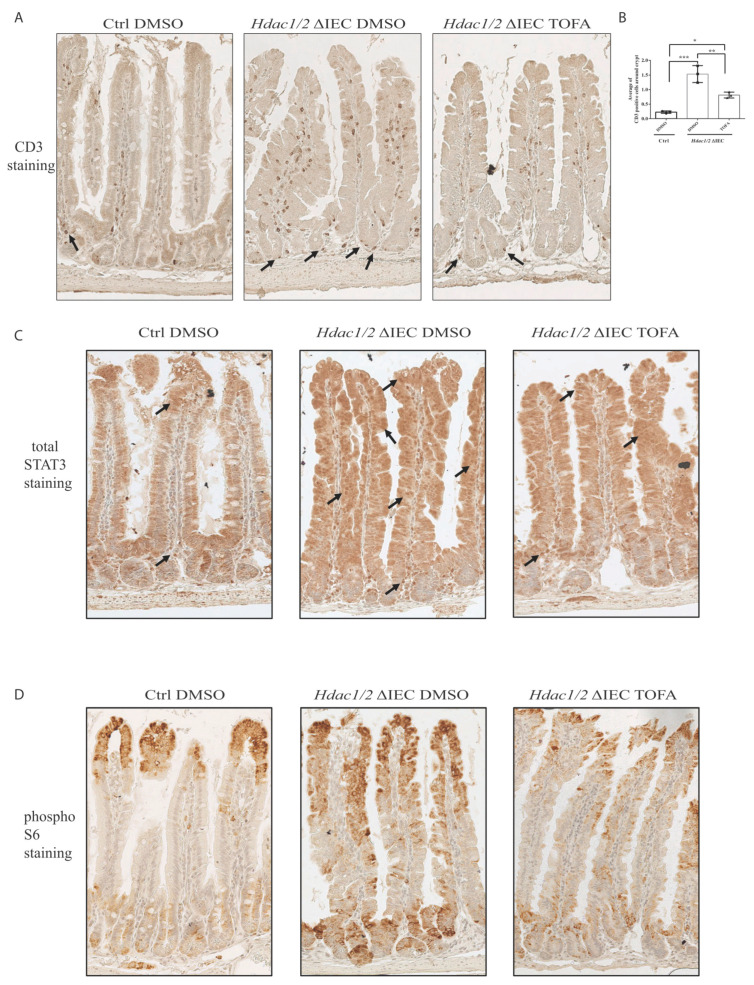
Tofacitinib treatment reduces T cell numbers and partially restores mTOR and JAK/STAT signaling pathways in villin-Cre *Hdac1* and *Hdac2* deficient mice. Jejunal tissue sections, from wild-type (Ctrl) as well as villin-Cre *Hdac1*^−/−^; *Hdac2*^−/−^ mice (*Hdac1/2* ΔIEC) treated without or with Tofacitinib (DMSO or TOFA) for five days, were stained with an antibody against CD3 for T cells. T cells are marked by black arrows (**A**). Average number of CD3+ cells around crypts is shown in (**B**) Results represent the mean ± SEM (* *p* ≤ 0.05; ** *p* ≤ 0.01; *** *p* ≤ 0.005). Jejunal tissue sections were stained with an antibody against STAT3 (**C**) and against phospho-S6 ribosomal protein (**D**). Magnification: 10×.

We had found previously increased immune T cell recruitment in the colon of villin-Cre dual *Hdac1*^−/−^; *Hdac2*^−/−^ mice [25]. As opposed to wild-type mice, we did observe more abundant immune T cells in mutant murine jejunal villi and bottom crypt regions, as assessed by CD3 antibody staining (Figure 7A). Increased CD3+ immune cell numbers correlate with intestinal inflammation observed in mutant mice. This could be a response to barrier defect and antimicrobial secreting Paneth cell loss. Treatment with Tofacitinib reduced the number of recruited T cells, notably in the surrounding crypt region, as observed with the decreased number of CD3+ cells around the crypt in treated mutant mice (Figure 7B).

We then determined the status of STAT3 activation in mutant mice treated without or with Tofacitinib, by analysing STAT3 nuclear localization by immunohistochemistry. In control mice, STAT3 was localized in the cytoplasm of IEC in the villi as well as in the top portion of the crypts (Figure 7C). Mutant jejunal epithelium displayed increased STAT3 nuclear staining along the crypt-villus axis. STAT3 labeled lamina propria cells were increased in the crypt region and the bottom of the villi (Figure 7C). Tofacitinib treatment reduced STAT3 nuclear staining, more prominently in the bottom of the villi. (Figure 7C). Thus, Tofacitinib treatment partially restored STAT3 nuclear levels to wild-type levels, in mutant mice. 

We then verified the status of mTOR pathway activation by staining with an antibody against phospho-S6 protein, a marker of mTOR activation. In control mice, we observed phospho-S6 staining restricted to two defined regions, namely in the top crypt and bottom villus region, and in the villus tip region (Figure 7D). This staining is associated with region-specific enterocyte functions. Indeed, while bottom villus region-located enterocytes are associated with biosynthetic efficiency and an antimicrobial pattern of expression, including Reg genes, enterocytes at the villus tips express cell adhesion and purine catabolism genes and are associated with an anti-inflammatory program [4]. Mutant mice displayed increased phospho-S6 staining which expanded in the middle villus regions (Figure 7D). While some phospho-S6 stained patches were maintained in middle villus sections, Tofacitinib treatment reduced overall phospho-S6 staining expansion in mutant mice. Moreover, it has been observed that mTOR inactivation in intestinal epithelial cells blocks Notch signalling, which in turn increases Paneth cell numbers [60]. In mutant mice, Paneth cell number increases after Tofacitinib treatment may indirectly stem from both mTOR and Notch inhibition. 

Thus, JAK/STAT inhibition in mutant mice leads to partial restoration of the epithelium, notably in the crypt, concomitant with Notch signaling inhibition, as exemplified by increased production of Paneth cells. Barrier and cell polarity are improved, as shown by restoration of baso-lateral CLDN3 expression and apico-basal organization, and with mTOR signaling recovery. In addition, mTOR signaling is partly recovered, as evidenced by recovery of phospho-S6 expression pattern in the epithelial mucosa. Our data show that JAK/STAT pathway is an important regulator of Notch and mTOR pathway activation in response to HDAC1 and HDAC2-dependent epigenetic IEC alterations. Tofacitinib-mediated inhibition of JAK-STAT signaling in IEC could well synergize with its anti-inflammatory role in immune cells [61], thereby impacting mucosal recovery during inflammation.

## Data Availability

The data supporting reported results can be found at: proteomeXchange Consortium through the PRIDE partner repository with the dataset identifier PXD (PXD022558) for proteomic data. RNA-Seq data have been deposited in the Gene Expression Omnibus database (GSE158522).

## Supplementary Materials

The following are available online at https://www.mdpi.com/2073-4409/10/2/224/s1. Figure S1: Enteroid growth is disrupted after *Hdac1* and *Hdac2* deletion. Representative micrographs of *Hdac1*^−/−^; *Hdac2*^−/−^ villin-Cre enteroids grown for 24 or 72 h (*n* = 3). Magnification: 5×. Figure S2: Villin-Cre *Hdac1* and *Hdac2* deficient mice are more sensitive to Notch pathway inhibition by DBZ. **A**. Jejunal tissue sections, from wild-type (Ctrl) as well as villin-Cre *Hdac1*^−/−^; *Hdac2*^−/−^ mice (*Hdac1/2* ΔIEC) treated without or with DBZ (DMSO or DBZ) for five days, were stained with hematoxylin and eosin (**A**) (Magnification: 10×). **B**. Wild-type (Ctrl), as well as villin-Cre *Hdac1*^−/−^; *Hdac2*^−/−^ mice treated without or with DBZ (DMSO or DBZ) for five days, were injected with BrdU for 2 h. Jejunal tissue sections were stained with an antibody against incorporated BrdU. Magnification: 10×. Table S1: List of upstream and downstream primers used for qPCR analysis. Table S2: List of antibodies used for immunofluorescence and Western blot analysis. Table S3: List of enriched *Hdac1* and *Hdac2* deleted jejunal intestinal epithelial cell mRNAs increased or decreased two-fold (fold-change: more than 2-fold and less than 0.5-fold). Table S4: List of enriched *Hdac1* and *Hdac2* deleted jejunal intestinal epithelial cell proteins increased or decreased by 50% (fold-change: more than 1.5-fold) and less than 0.75-fold). Table S5: List of enriched *Hdac1* and *Hdac2* deleted jejunal intestinal epithelial cell Disease and Bio functions (z-score) shared between RNA-seq and proteomics data. Table S6: List of enriched *Hdac1* and *Hdac2* deleted jejunal intestinal epithelial cell upstream regulators identified by IPA analysis from RNA-seq data and from proteomics data.
