# Supplementary material for: JAK-STAT Pathway Inhibition Partially Restores Intestinal Homeostasis in Hdac1- and Hdac2-Intestinal Epithelial Cell-Deficient Mice
Source: Cells. 2021 Jan 23;10(2):224. doi: 10.3390/cells10020224 (PMC7911100; doi:10.3390/cells10020224)

Supplementary figure 2

A- **HE staining**

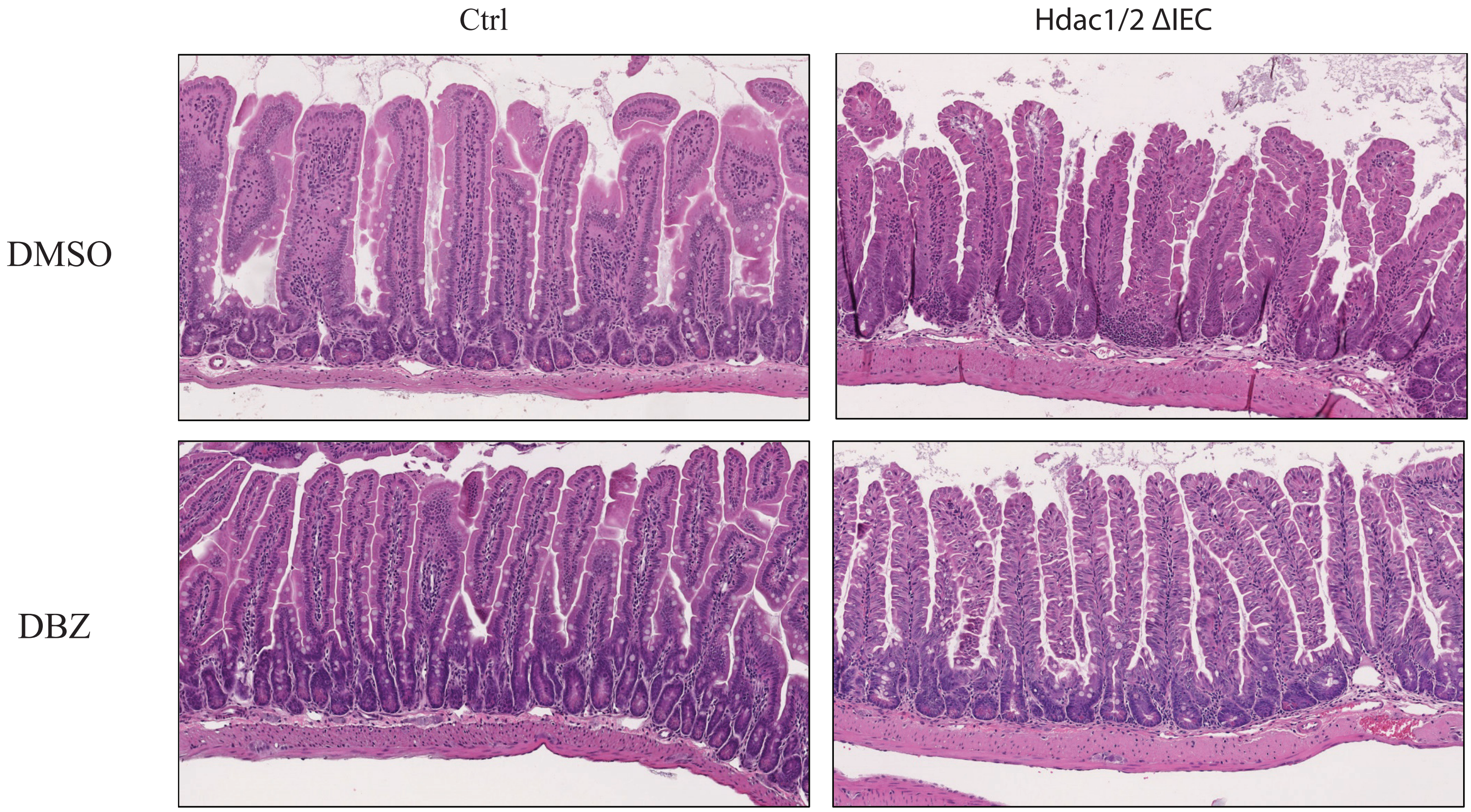

B- **BrdU staining (2 h)**

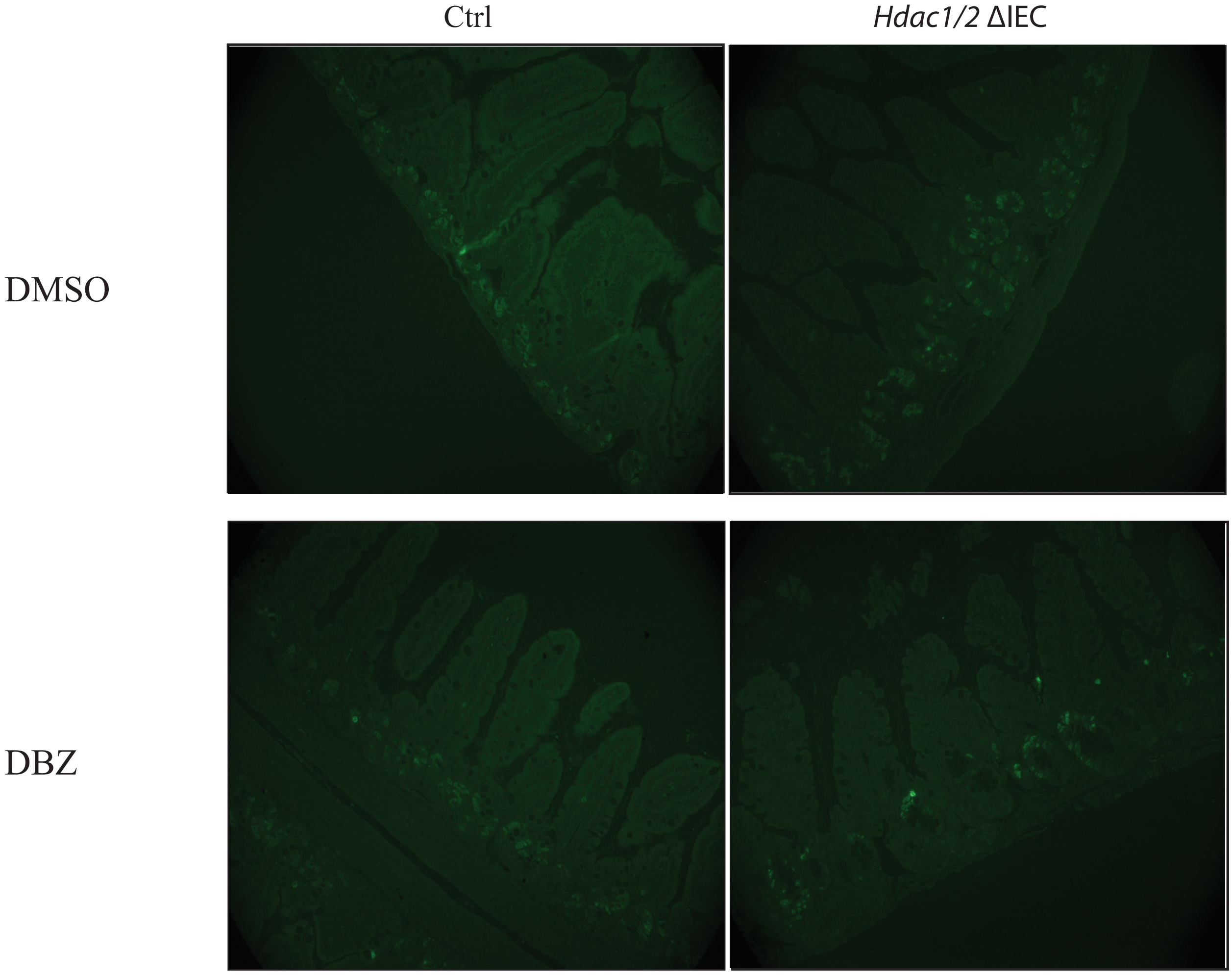

Supplement: Supplementary file 1 [file cells-10-00224-s001.zip › Suppl figure S2.pdf]
